# Memoir on the Contagious Disease Which Visited the Communes of Duhort, Buanes, Bahus, &c. Last Year; with a Sketch of That Which Prevailed in the Hospitals Attached to the Grand Army in Spain; and on the Treatment and Preservative Regimen Most Proper to Be Pursued

**Published:** 1810-01

**Authors:** M. Dupin

**Affiliations:** Chief Physician to the Military Hospital of St. Sever; and Government Physician for Epidemic Diseases


*Memoir on the Contagious Disease which visited the Communes of Duhort, Buanes, Bahus, &c. last Year; with a Sketch of that which prevailed in the Hospitals attached to the Grand Army in Spain; and on the Treatment and preservative Regimen most proper to be pursued.* By M. Dupin, M. D. Chief Physician to the Military Hospital of St. Sever; and Government Physician for Epidemic Diseases.*

*Aliquid semper ad communem utilitatem afferendum.*

I Am about to offer an account of the contagious disease which prevailed last year in the communes adjoining my residence, and which generally ended in the death of the persons attacked. I shall add an account of the contagion, which lately broke out in the military hospitals in Spain, and among the inhabitants of the country, which, last, was extremely analogous to the disease in the army. My only wish is to be correct, as to facts, keeping constantly in view the sentiment of the philosopher and physician. "If truth is only a point of honour with the bulk of mankind; with the physician it ought to be peculiarly sacred and inviolable." Finally, I shall succinctly trace from actual observation, and not from hypothesis, the proximate as well as the remote causes of the disease. It may be thought as the epidemic has ceased to exercise its ravages in the communes where I witnessed it, that this memoir is now superfluous. There would be some weight in this objection, if experience had not demonstrated that epidemics, like the above, are apt to return, particularly to us who are in the vicinity of a large army, in which it prevails with more or less severity, and from which we are likely to receive infected individuals. Above all, I wish it to be understood, that I do not write for the sake of my brethren physicians, who are not likely to go wrong, but country surgeons are generally the first called in to patients of the above description, and on the first treatment of the disease the issue frequently depends.

The contagious fever first alluded to in the title of this memoir, chiefly prevailed in the communes of Duhort, Buanes, Bahus, &c.; and admitting that there was a difference in some of the symptoms, as they manifested themselves in the various communes, we ought, nevertheless,

---

* Journal de Medicine.

to class the disease, according to the general characteristics it bears; for a treatment which suits one epidemic, is certainly applicable (under certain modifications dictated by circumstances) to all epidemics, bearing the same character in their leading symptoms.

The terror and alarm which never fail to accompany the ravages of epidemic diseases, are among the primary causes of their propagation. The blind prejudice which seizes the vulgar, perverts their understanding, and proves the predisposing cause of the disorder.

It would be very important to show that these diseases have no contagious character, and that it is only terror, prejudice, or negligence, which renders them so fatal. In the country, there exist no precise ideas respecting contagious diseases, which are most frequently complicated, and sometimes disguised under symptoms apparently foreign to them. A long experience in the armies, and in this part of the country, has convinced me that anomalous fevers, commonly called malignant epidemics, as well as almost all acute diseases, generally become unmanageable when we neglect to attack them in their commencement; the only period at which we can effectually restrain their violence, and mitigate their symptoms.

The disease, of which I am to give the history, exhibited in the course of its different stages, singularities and phenomena, which excited my particular attention. I shall confine myself to what I saw and observed in a single family, all the members of which were successively attacked with the most distressing symptoms. Let me previously state what occurred before I was sent for. The wife of Labarthe, called Ann Boueilles, in the commune of Bahus, 55 years of age, went to Buanes in the beginning of January 1808, to attend the husband of her sister, who laboured under the disease then raging in the commune. The brother in law was cured, but his wife, sister to Ann Boueilles, died. Labarthe's wife returned to her family, having absorbed the deleterious poison, was confined to her bed, and communicated the disease previous to her death, which soon happened, to her husband, who was upwards of 60 years old; to her son Peter, aged 30; to her daughter in law, Jane; to her son John, aged 25; to a nephew, who was in the house; and to a shepherd boy, aged between 12 and 13. M. Durrieu, receiver of the taxes at St. Sever, and proprietor of the farm occupied by this unfortunate family, four individuals of which had already received the last sacrament, requested me to visit them. He

He described them as abandoned by their relatives and friends, and condemned to inevitable death. I instantly proceeded to the scene of distress, accompanied by M. Latour, the officer of health at Mont Gaillard, who being convinced of the desperate nature of the disease, considered my visit as superfluous.

Having entered the house, I made a large fire: my attention was then directed to the apartments and beds of the patients. I opened all their curtains to admit the fresh air, inundated them with fumes of vinegar, and ordered fomentations of vinegar and water, for the upper and lower extremities, every two hours.

Not having a great stock of pharmaceutical preparations at my command, I prepared a remedy chiefly composed of theriaca and good wine. The father of the family in particular was in the last agonies. I administered the same mixture to all of them, and having called in the neighbours, assured them there was no danger. M. Durieu, on my application, sent me an abundant supply of vinegar and good wine, with some additional attendance; in short, by assiduity night and day, and by administering large quantities of the above medicine for upwards of a month, the whole of the family was restored to health; the confidence of their neighbours returned, their fears were banished, and the commune was preserved from the further ravages of this alarming pestilential malady.

The disease which forms the subject of this memoir, and which has been described by the appellation of the jail fever by Pringle, the malignant putrid fever by Huxham, the malignant catarrhal fever by Eller, and which has been so successfully elucidated by Lind, Monro, and Sydenham, and now generally known by the name of *ataxic fever*, seemed to me to be true acute nervous fever, rarely verminous, and still more rarely complicated with inflammatory diathesis. The primary symptoms, as well as those of the subsequent stages of the disease, exhibit this character in a very decided manner.

Diseases which attack a great number of patients at the same time, and in the same country, and consequently bear the character of epidemics, must depend on an universal and common agent, but accidentally introduced to the inhabitants of this or that district. Now we only find this universal and common agent in our food, or in the air we breathe, two things common and necessary to all mankind. In the present instance, the disease seems to have been in a great measure excited by the physical qualities of the

 air, air. After a very damp autumn, the succeeding winter renders all the actions of nature torpid; while the piercing cold that prevailed with so much constancy, must have prevented a free perspiration. Experience proves that cold northern blasts become hurtful, when they exceed certain limits. Fear and other mental affections contributed, in addition to material causes, in the developement of the disease. It does not appear that any universally endemic cause existed; diarrhœa did not precede the disease, as we find it does, at this moment, in the hospitals attached to the army of Spain, and among such of the inhabitants as are infected with the same contagion.

Hitherto, a blind empiricism has been the only guide of medical men, in the treatment of a species of disease which has been branded with a malignant character, not on account of the formidable effects it produced, but because these effects were ascribed to certain deleterious principles, the origin and nature of which they never attempted to discover.

Dissections of a great number of dead bodies, and the most profound observations, at length, concurred to throw some light on the inextricable labyrinth of symptoms which attend this species of disease; a method of treatment established on the most solid foundations, has been constantly attended with success, in the hands of all who have resorted to it. Nature must be followed step by step; when we are once acquainted with the chain of facts which occur in practice, they must be compared with each other, and considered with regard to the age and sex; the temperaments of the patients, as well as the climate.

Although the disease varies much in its degrees of intensity, and the rapidity of its periods; although in children and adults of a feeble constitution, these periods were less rapid, and less mortal, than in persons of plethoric habits; although we have seen individuals suddenly attacked through the medium of contagion, and without any apparent prodromus,—we have nevertheless, in general, ascertained three very distinct degrees in this disease.

The first was announced by spontaneous lassitudes; by head-ache, which was the most constant symptom, and generally very violent, but sometimes confined to a painful sensation of heaviness or stupor, with no fixed seat, being sometimes in the lower and at other times in the back or upper part of the head; by want of sleep, or laborious and oppressive slumbers; numbness of the limbs; changes from heat to cold; pulse small, somewhat hard, and and unequal; tongue white, dry, and furred; frequently also there were retchings without any sign of swelling or tension of the abdomen; the eyes in some patients were brilliant and fixed, in others dull. The second degree was marked by a continual delirium, mostly of a tranquil kind, but sometimes furious; and by spasmodic twitches, &c. The third degree exhibited subsultus tendinum, as the characteristic symptom; deafness; drowsiness; an extraordinary prostration of strength; small pulse; hiccough; colliquative sweats; petechiæ; tongue black and dry; and lastly, a lengthening of the countenance, which is always a sign of extreme danger, while a contrary appearance is the most certain sign of convalescence; marks of putridity were also present in the third degree.

The general duration of the disease was twenty-one days; after this period the most alarming symptoms ceased. Convalescence was tedious, however, and relapses were frequent. In the highest degree of the fever, two exacerbations were uniformly remarked during the day; one began in the morning, and was at its height at noon; the other began towards evening, and was at its height about midnight; the urine was slimy during the whole of the disease; it was thicker in proportion as the disease was more dangerous; the danger was imminent when in the highest stage of the fever, the urine *suddenly* became clear and limpid; whereas the most unerring sign of an approaching recovery was, when the urine became gradually clearer, and deposited less sediment.

My treatment of the disease chiefly consisted in keeping up and restoring the strength by the general application, in suitable proportion, of volatile stimulants, such as camphor, musk, ether, valerian, Virginian snake root, ammonia, quinquina, and above all, good wine and theriaca; it being requisite, in order to obtain a complete cure, not only to recruit the physical strength, but also to combat the deleterious cause of the disease.

If we consult the most eminent writers on contagious diseases, such as Sydenham, Huxham, Pringle, Lind, and Monro, we find that they considered them under the two following aspects; viz. as depending on miasmata, which floats about in the stomach or cellular texture, or as fixed in these parts, having induced some deep seated putrid injury. It is in the first state of the disease that we have seen the antimonial tartrite of potash used with the greatest efficacy; this remedy seems to us to act less in evacuating the stomach, than in producing an uninterrupted and salu-

 tary tary perspiration; not only has it the advantage of expelling the injurious matter contained in the stomach, and thus occasionally combating the cause of contagion; but it also rouses the vital energies as a consequence of the action impressed on the whole nervous system. We have also, on the present occasion, in imitation of Sydenham, subjected the patients to a sudorific and antispasmodic treatment. I have seen perspirations, whether caused by nature or by artificial means, bring the diseases to a close at this period. Another remedy, of evident success in the same case, was a blister applied between the shoulders; the delirium, head-ache, and flying pains, which resisted the effects of a vomit, yielded to this application.

In the second state, and when the contagious miasmata had decidedly effected a septic injury, or produced a paroxysm, we have successfully combined antispasmodics with anti-septics. The mixture which succeeded best was the bark, in combination with camphor, serpentaria, valerian, &c. But the remedy which most decidedly completed the cure of these cases, as well as in others, attended by my colleague M. Dufour in the communes of Buanes, Bahus, &c. was topical bleeding by means of leeches; a practice which combines the double advantage of evacuating the part immediately diseased, and relieving the interior of the head, whilst it weakens the patient less than general bleeding. This last method of evacuation, in consequence of the total prostration it occasioned, we found as injurious and dangerous as purgatives, which must be cautiously avoided. With the same view, we applied fomentations of warm water, vinegar, and muriate of soda to the lower extremities, renewing them every two hours until the head was relieved. We did not neglect the use of glisters to keep the belly open.

The cleanliness of the patients, and every thing about them, constantly occupied our attention; the bed curtains were continually open to admit fresh air, and the fumigations prescribed by Guyton de Morveau, were carefully performed.

One of the symptoms, which particularly manifested itself among the military in Spain and the inhabitants who contracted the contagion, was, and still continues to be, a serous diarrhœa, which, when it has become moderate, and seems to have diminished the disease, ought not to be stopped; but when it is too abundant, and visibly weakens the patient, I have successfully administered boluses made of of two parts of ipecacuanha and one of opium; the arnica montana in decoction or powder, blisters applied only as rubefacients, without destroying the epidermis. I have also administered wine in large doses at this period, as a strengthener and antiseptic: we know how advantageous this practice was when resorted to by Sir John Pringle. Asclepiades says, that wine in its effects on these diseases, disputes pre-eminence with the power of the gods.

In the more advanced stages of the disease, i. e. in the third degree, I increased the dose of the internal remedies; varying and combining them according to circumstances. I employed spirituous aromatic lotions, and had recourse to external stimulants in their greatest possible energy, applying them to almost every part of the body to recall the fleeting spark of life. With a view to diminish the interior heat, I applied vesicatories of cantharides, much more rarely than sinapisms, because the former acting more slowly, often produced bad conditioned ulcers, chiefly when applied to the legs. The old man, who was the husband of Madame Labarthe, presented a striking example of this fact; the wounds in his legs, from the effects of vesicatories applied before I was consulted, became gangrenous and lost all sensation, giving out a fœtid and cadaverous sanies. I ordered scarifications, more or less deep, according to the extent of the gangrene, bathing the wounds with camphorated water; and in proportion as the disease was subdued by the medicines taken internally, the wounds assumed a better appearance, and at length they were cicatrized, attended, however, with a great loss of substance.

My friend, M. Dufour, witnessed a case of this kind, which deserves to be mentioned. A woman, of the name of Anne Phillippe, whom he visited at Duhort, by desire of the sub-prefect, lost all the muscles and cartilages of her nose, which was ulcerated, became gangrenous, and fell off in sphaceli, leaving all the nasal bones exposed. The patient, notwithstanding this unfortunate event, recovered by M. Dufour's skill.

The warm bath is, in my opinion, salutary beyond measure; no application operated so speedily in restoring the strength of the patient, diminishing the rapidity, and re-establishing the equality of the pulse; in allaying spasms, convulsions, delirium, and pains in the lower belly; and, lastly, in assisting a good perspiration, and regularly developing the other secretions. These baths would be still more efficacious, if they were rendered aromatic by the addition of camomile flowers, rosemary, lavender, thyme, and sweet marjorum, two ounces of each at a time; the heat of the bath should never exceed 27° or 28° of Reaumur, and the patients ought not to remain in it longer than eight minutes. I have also applied with success a bag of aromatics, boiled in wine, to the region of the stomach.

Let us now attend to the means of preservation. But first let us remark with Hippocrates, that it is very difficult to resist the action of the deleterious causes with which we are surrounded. *Plerumque hominis natura universi potestatem non superat.* Man, like all other organised beings, is under the controul of external objects, with which he is compelled, by his nature, to keep up a connection; he is subject to all the alterations which the elements can effect; as long as he breathes, he is exposed to the influence of the atmosphere.

We may, however, offer a word on the most proper regimen, and on the state of mind best adapted to resist contagion; and afterwards speak of the means to be employed to preserve us from infection.

All sudden and abrupt changes are prejudicial, when an epidemic or contagious disease prevails. We would not advise those, whose only nourishment is milk, suddenly to change it for animal food and spirituous liquors. The discharge of wind upward and downward, which the patients continually void, and the worms which sometimes render the disease more complicated, seem to forbid the use of vegetables. We would wish, therefore, that fruits, such as apples, pears, figs, &c. should be prohibited. The farinæ seem also improper, particularly for children. The tetradynamic plants, and the alliaceæ, such as radishes, water-cresses, turnips, carrots, onions, asparagus, garlic, &c. with a moderate use of animal food and wine, form the most proper nourishment. While this regimen is pursued, all excesses must be avoided, particularly of cold or humidity, which materially predispose the constitution for the disease. The mind ought to be kept calm and tranquil; fear, discontent, and all the enervating passions, ought to be dismissed by all possible means; and particularly by proper amusements, or a moderate use of good wine, &c.

In order to prevent the propagation of the disease, all communication ought to be interdicted between the infected and the healthy; the hospitals should be removed from the central part of the town, and guards stationed to prevent prevent the patients from leaving the hospitals until they are completely well, and until their linen and persons have been fumigated. The sick ought to be kept very clean, and their bed-clothes frequently changed. We have always found the disease more serious, when the patients were deprived of the use of fire. Wagler and Rœderer made a similar observation at Göttingen, and Lind in Haslar Hospital. The stools of the patients must be quickly removed: it would be even prudent to bury them under ground. The linen ought to be soaked for some time in cold water, and afterwards passed through a lye: without this precaution, the evaporation of the water might carry up the contagious miasmata, and thus propagate the disease. The air of the apartment should be frequently renewed; and not satisfying ourselves with the introduction of fresh air, we should diffuse the vapours of oxy-muriatic gas, or any similar substance, in the sick room, taking care not to incommode the breathing of the patient. The furniture and clothes in the apartment ought also to undergo similar fumigations.

